# Metformin a Potential Pharmacological Strategy in Late Onset Alzheimer’s Disease Treatment

**DOI:** 10.3390/ph14090890

**Published:** 2021-09-01

**Authors:** Saghar Rabiei Poor, Miren Ettcheto, Amanda Cano, Elena Sanchez-Lopez, Patricia Regina Manzine, Jordi Olloquequi, Antoni Camins, Mohammad Javan

**Affiliations:** 1 Department of Physiology, Faculty of Medical Sciences, Tarbiat Modares University, Tehran 14117-13116, Iran; saghar.rabii@gmail.com; 2 Institute for Brain and Cognition, Tarbiat Modares University, Tehran 14117-13116, Iran; 3 Department of Pharmacology, Toxicology and Therapeutic Chemistry, Faculty of Pharmacy and Food Sciences, Institut de Neurociències, University of Barcelona, 08028 Barcelona, Spain; mirenettcheto@ub.edu (M.E.); patricia_manzine@yahoo.com.br (P.R.M.); 4 Biomedical Research Networking Centre in Neurodegenerative Diseases (CIBERNED), 08028 Madrid, Spain; acanofernandez@ub.edu (A.C.); esanchezlopez@ub.edu (E.S.-L.); 5 Ace Alzheimer Center Barcelona, Universitat Internacional de Catalunya (UIC), 08028 Barcelona, Spain; 6 Institute of Nanoscience and Nanotechnology (IN2UB), 08028 Barcelona, Spain; 7 Department of Pharmacy, Pharmaceutical Technology and Physical Chemistry, Faculty of Pharmacy and Food Sciences, University of Barcelona, 08028 Barcelona, Spain; 8 Department of Gerontology, Federal University of São Carlos (UFSCar), São Carlos 13565-905, Brazil; 9 Laboratory of Cellular and Molecular Pathology, Institute of Biomedical Sciences, Faculty of Health Sciences, Universidad Autónoma de Chile, Talca 3467987, Chile; jolloquequig@uautonoma.cl; 10 Department of Brain and Cognitive Sciences, Cell Science Research Center, Royan Institute for Stem Cell Biology and Technology, ACECR, Tehran 14117-13116, Iran

**Keywords:** Alzheimer’s disease, diabetes mellitus, metformin, insulin resistance, beta amyloid, tau protein hyperphosphorylation, AMP activated protein kinase (AMPK)

## Abstract

Alzheimer’s disease (AD) is one of the most devastating brain disorders. Currently, there are no effective treatments to stop the disease progression and it is becoming a major public health concern. Several risk factors are involved in the progression of AD, modifying neuronal circuits and brain cognition, and eventually leading to neuronal death. Among them, obesity and type 2 diabetes mellitus (T2DM) have attracted increasing attention, since brain insulin resistance can contribute to neurodegeneration. Consequently, AD has been referred to “type 3 diabetes” and antidiabetic medications such as intranasal insulin, glitazones, metformin or liraglutide are being tested as possible alternatives. Metformin, a first line antihyperglycemic medication, is a 5′-adenosine monophosphate (AMP)-activated protein kinase (AMPK) activator hypothesized to act as a geroprotective agent. However, studies on its association with age-related cognitive decline have shown controversial results with positive and negative findings. In spite of this, metformin shows positive benefits such as anti-inflammatory effects, accelerated neurogenesis, strengthened memory, and prolonged life expectancy. Moreover, it has been recently demonstrated that metformin enhances synaptophysin, sirtuin-1, AMPK, and brain-derived neuronal factor (BDNF) immunoreactivity, which are essential markers of plasticity. The present review discusses the numerous studies which have explored (1) the neuropathological hallmarks of AD, (2) association of type 2 diabetes with AD, and (3) the potential therapeutic effects of metformin on AD and preclinical models.

## 1. Introduction

Currently, there are 44 million people dealing with dementia, being the second leading cause of death in people aged 70 and over [1,2,3,4]. Alzheimer’s disease (AD) is the most prevalent form of dementia [1] and it is also one of the most significant causes of morbidity and mortality in the elderly population worldwide [3]. AD prevalence is estimated to reach 115 million by 2050 due to an increased ageing population pattern, unless novel drugs are available to slow or cure this disease [2,3,4]. Neuropathological modifications of AD, such as tau hyperphosphorylation and Aβ toxicity, led to the main current hypothesis trying to explain neuronal and synapse loss, associated with cognitive and memory impairment [2,3,4,5,6].

Since the neurodegenerative process of AD is closely related to the aging process, it has also been called the late onset AD (LOAD). LOAD lacks a clear origin, and it is mainly found in patients over 65 years old. These patients comprise 95% of AD cases, although about 1% are attributed to autosomal dominant mutations in amyloid metabolism-related genes, e.g., beta amyloid precursor protein (βAPP-chromosome 21), presenilin 1 (PS1-chromosome 14), and presenilin 2 genes (PS2-chromosome 1) [6].

In spite AD or dementia is often related with the geriatric community, AD may also affect younger adults. Early-onset AD (EOAD) is referred to those affecting people who are diagnosed before 65 years old (between 30 and 65). As outlined by McMurtray and colleagues, early-onset dementia patients account for 20% to 34% of AD cases in most trials [7]. Three gene mutations playing a key role in EOAD have been identified: APP, PS1, and PS2. PS1 and PS2 are two proteins that constitute the catalytic core of γ-secretase. These gene mutations—including APP, PS1, and PS2—belong to the familial form of AD and have been shown to increase the development of amyloid-β (Aβ) leading to an increase in the Aβ1–42/Aβ1–40 ratio, hence favoring the formation of senile plaques [1]. A combination of genetic and environmental factors and lifestyle elements play a significant role in LOAD, which does not have a clear etiology and it is considered multifactorial [7].

While LOAD has not a well-established etiology, two of the most relevant genes conferring a significantly risk factor are the E4 apolipoprotein allele (APOEε4) and the triggering receptor expressed on myeloid cells 2 (TREM2) [8,9,10]. The ε4 allele of the APOE gene codes for the main apolipoprotein of the CNS, whose functions are lipid transport and neuron homeostasis [8,9,10,11,12]. It has been reported that one copy of the ε4 allele in APOE increases LOAD risk by 3~4-fold [10,11]. Its mutation results in a higher lipid binding capacity of APOE4, and it is associated with a less efficient clearance of Aβ and an increase in pathological changes responsible for cognitive decline [10]. On the contrary, carriers of the APOEε2 allele have two times less risk of suffering from LOAD than non-carriers, being considered a protective genetic factor against this disease [12]. There are significant clinical and basic evidence that shows early driving amyloid pathology in the brains of APOEε4 carriers [8,9,10]. Furthermore, in several brain homeostatic pathways, including lipid transfer, synaptic integrity and plasticity, glucose metabolism and cerebrovascular activity, APOE4 is either pathogenic or a decreasing factor of performance [8,9,10,11]. In turn, TREM2 is a very abundant receptor on the surface of microglia and plays an important role in its activation and regulation [13,14]. Certain mutations can condition the affinity of TREM2 for its ligands, decreasing phagocytosis of Aβ peptide by microglia and promoting a systemic inflammatory response. Thus, TREM2 deficiency is involved in the development of LOAD due to an insufficient microglial function. In addition, TREM2 regulates the function of microglia in LOAD and other neurodegenerative diseases, and also participates in inflammatory responses and metabolism, either alone or in close association with other molecules, such as APOE [15].

Type two diabetes mellitus (T2DM) and LOAD have become worldwide pandemics, with recent projections indicating that they will get worse in the coming decades. In this respect, obesity, T2DM and associated comorbidities have been described to be involved in the development of LOAD [14]. Thus, LOAD has been recently described as a “metabolic disease”, related with the inefficient utilization of glucose by the brain and associated with insulin resistance and chronic mild inflammation in the brain [15,16,17,18,19,20]. Likewise, and due to the insulin resistance generated in the brain, LOAD has been also referred to as “type 3 diabetes” [19,20]. Like T2DM, which is characterized by a decreased ability of peripheral tissues to metabolize glucose, in LOAD the decreased ability of brain to metabolize glucose (brain glucose hypometabolism) could contribute to the neurodegenerative process, together with the classical neuropathological LOAD hallmarks, such as Aβ deposits and hyperphosphorylated tau (p-tau) in neurofibrillary tangles (NFTs) [19,20].

Preclinical research studies and clinical and epidemiological trials reported that T2DM has been related not only to the development, but also to the progression of LOAD. A retrospective study of 20 prospective clinical trials concluded that the prevalence of LOAD in patients with T2DM was 56% greater than in people without diabetes [21]. Brain insulin resistance, decreased insulin signaling, inflammation, hyperglycemia, vascular alterations, hypoglycemic events, and impaired amyloid metabolism are proposed causes for this relationship [9]. Consequently, it has been shown that both T2DM and LOAD possess multifactorial risk profiles and a wide variety of molecular connections. The intersection between the molecular pathways of these two diseases could give birth to the appearance of the cognitive anomalies of LOAD patients with underlying T2DM [22,23,24,25].

## 2. Type 2 Diabetes Mellitus Related with Alzheimer’s Disease

T2DM is a widely known chronic metabolic condition characterized by high levels of blood glucose and insulin resistance [24,25,26]. Epidemiological findings indicate that, relative to normal people, some diabetic patients have an elevated chance of developing dementia. Hence, in the ‘Rotterdam Study’, Ott and colleagues were the first who reported the potential connection between both pathologies, disclosing that diabetes significantly increased the risk of dementia [27,28]. Thus, it was suggested that LOAD can be viewed as a metabolic disorder because brain of LOAD patients showed several features in common with compromised insulin signaling pathways [21]. Additionally, clinical and epidemiological studies have confirmed this association, demonstrating that the alteration of metabolic parameters, such as hyperglycemia and hyperinsulinemia, are positively correlated with the development of LOAD neuropathology [29]. In this sense, the ‘Hisayama Study’ reported an association between diabetes plus APOEε4 and Aβ plaques, but not with neurofibrillary tangles formation [30]. In turn, in a study performed in cognitively healthy middle-aged adults enrolled in the ‘Wisconsin Registry for Alzheimer’s Prevention study’, Willette and colleagues reported an association between brain insulin resistance (BIR) and Aβ brain deposition in LOAD patients, given support to the hypothesis that BIR is a risk factor in the early stages of LOAD [31,32]. Therefore, this study concluded that BIR is a modifiable risk factor during the preclinical stage of the pathology, which opened a new therapeutic window for the design of new strategies focused on the prevention of LOAD [31]. Likewise, some reports conclude that, under certain conditions such as metabolic disorders, the body’s diabetic status will enhance the occurrence of LOAD by disrupting the transfer of glucose into the brain and decreasing its metabolism [21]. Overall, impaired insulin signaling pathway is associated with metabolic disturbances such as glucose/lipid metabolism, protein modifications, mitochondrial dysfunction, and oxidative stress. In addition, BIR can exacerbate Aβ accumulation, increase tau hyperphosphorylation, devastate glucose transportation and energy metabolism, and impair hippocampal framework pathways [18,19,30]. Besides, it has been demonstrated that insulin has many positive effects on the brain, including synaptic trophic effects and dendritic spine development promotion [33,34,35]. Although the evidence of a correlation between diabetes and neurodegenerative pathology in LOAD is mixed, some human postmortem findings suggest a link between brain insulin tolerance and increased LOAD pathology, including increased Aβ deposition [35,36,37,38,39]. Moreover, as we have already mentioned above, several clinical studies have discovered that patients with diabetes have considerable levels of Aβ and p-tau in cerebrospinal fluid as well as lower scores of cognition [40,41,42,43,44]. However, evidence from pre-clinical and limited clinical trials indicate that insulin and agents that promote insulin signaling could reduce neuropathology and boost cognition in diabetes and LOAD [41,42].

The most common hypothesis recognizes Aβ as the main cause of LOAD [43,44,45,46,47,48,49]. This starts with amyloid beta precursor protein (APP), which breaks down to release Aβ due to the activation of a whole series of enzymes of the amyloidogenic cascade, such as β and γ secretases. This increases the levels of harmful Aβ 1-42 [46,47,48], enabling fibril production of extracellularly deposits that destroy neurons and organize classical senile plaques [46]. Hence, the initial amyloidogenic hypothesis suggested that amyloid plaques or insoluble amyloid fibrils were responsible for the loss of synapses since amyloid plaques are found in postmortem brains of LOAD patients and in preclinical models of AD that present cognitive deficits [46,47,48,49]. Although mice models generally develop amyloid plaques, they also show synaptic dysfunction and cognitive deficits prior to plaque formation. In this way, this hypothesis has been challenged and it is now accepted that soluble Aβ oligomers, also known as Aβ-derived diffusible ligands (ADDL), would be the powerful neurotoxins of the central nervous system (CNS) that accumulate in the brain in LOAD [46,47]. Based on these observations, Aβ toxicity is mediated not only by insoluble amyloid fibrils but also by ADDL, and synaptic failure is likely to be one of the earliest events in the pathogenesis of AD [46]. Moreover, it has been shown that ADDL correlate better with the disease severity (cognitive decline) than with the accumulation of insoluble Aβ peptides into plaques triggering AD pathophysiology [46,47]. Likewise, previous studies have demonstrated that ADDL are able to impair the function of insulin receptor in brain (detected at dendritic level in synapses), inducing an intracellular localization of this receptor which takes it away from the neuronal surface of dendrites. Therefore, this process is associated with a decrease of glutamatergic neurotransmission [46,47,48,49].

Furthermore, Aβ is linked to both neuronal oxidative stress and mitochondrial dysfunction [47]. Unfolded protein response or endoplasmic reticulum stress are also involved in the development of Aβ neuronal cell damage, also being closely correlated with the pathology of tau protein. Through the raise of Aβ pathology, these metabolic agents could increase the occurrence of LOAD in diabetic patients [50].

Apart from the effects of Aβ oligomers at the brain, a very interesting point is the role of plasma Aβ oligomers on peripheral IR. In line with this concept, it has been reported that these oligomers also have an inhibitory effect on the peripheral insulin signaling pathway through different mechanisms mediated by oxidative stress and inflammatory responses, hence demonstrating that Aβ oligomers modify peripheral glucose metabolism through multiple ways [51,52,53,54]. Thus, Aβ oligomers have important effects on the systemic metabolism of glucose, where insulin is critical for proper glucose homeostasis through effects on the liver, skeletal muscle, and adipose tissue [41,55]. Therefore, these studies give support to the hypothesis that LOAD is a metabolic disease in which Aβ oligomers production in the brain play a key role in peripheral metabolism alteration.

One of the characteristics of LOAD is the brain hypometabolism that is due to a decrease in glucose uptake. The drop in brain glucose levels is mainly related to a reduced glucose uptake associated with the decreased expression of glucose transporters in neurons, mainly GLUT1 and GLUT3 [56]. Therefore, it has been suggested that increasing glucose transport to neurons, for example with antidiabetic drugs such as metformin, may be a therapeutic approach in AD [56]. Likewise, alteration of different brain pathways associated with T2DM has been reported in LOAD patients. In LOAD, as we have already discussed, it has been demonstrated that an alteration of IR levels can affect the cognitive process due to synaptic impairment, in addition to increasing oxidative stress, thus favoring mitochondrial dysfunction that ultimately leads to neuronal apoptosis [38,39,40,41,42,43,44].

Chronic hyperglycemia can be responsible for the appearance of diabetic complications since it can generate glycolipotoxicity. In this way, hyperglycemia generates advanced glycation end products (AGEs) that are also a crucial link between diabetes and LOAD [48,49,50,51,52,53]. It has been proposed that an increase in AGE levels in the brain may be directly related to cognitive dysfunction in LOAD patients. For this reason, AGEs can contribute to LOAD by promoting the formation of fibrillar tangles and amyloid plaques, which are the main neuropathological characteristics of LOAD, in addition to increasing the cytotoxicity of Aβ [16,17,18,19,20]. AGEs induce the expression of their receptor, RAGE, which is also a putative receptor for Aβ [16,17,18]. Previous studies have shown that RAGE levels are increased in various types of LOAD brain cells. For example, glial cells of the brain show elevated levels of RAGE and, furthermore, a colocalization of RAGE with intracellular Aβ and tau has been observed in LOAD patients.

Besides, the key enzymes glycogen synthase kinase 3β (GSK3β) and insulin degrading enzyme (IDE) are altered in bothT2DM and LOAD, being a potential link between the two diseases. GSK3β is regulated by the insulin receptor and its activation in LOAD favors the phosphorylation of tau protein and the formation of neurofibrillary tangles [40,41]. IDE plays a key role in the metabolism and elimination of Aβ and also insulin. IDE degrades both insulin and the Aβ peptide, however, insulin binds to IDE with higher affinity. Therefore, in both diseases, T2DM and LOAD hyperinsulinemia sequesters IDE, presenting a greater affinity for insulin than for Aβ and, for this reason, it ends up facilitating the accumulation of Aβ levels and increasing the risk of LOAD [55].

Supporting this metabolic hypothesis of LOAD, a preclinical study with a neuron-specific human BACE1 knock-in mouse model (PLB4) conducted by Plucińska and colleagues demonstrated that the brain BACE1 overexpression by itself increased the risk of peripheral T2DM [56]. Therefore, this study suggested that LOAD progression can promote T2DM comorbidities in mice, independently of the classical obesogenic process, which could be a potential link between T2DM and LOAD.

As we have already mentioned, tau is the other main biomarker related to the neurodegenerative process in LOAD [1]. Tau is a hydrophobic protein involved in the neuronal stabilization of microtubules and axonal transport. According to the “tau hypothesis”, tau protein’s dysfunction leads to form NFTs [4,6,57]. Specifically, tau hyperphosphorylation interrupts its connection with microtubules, which disrupt the entire microtubules assembly [1,4,6,58,59,60,61].

In this sense, brain insulin has been shown to play a key role in the regulation of tau phosphorylation through the activation of its receptor located in the brain. Indeed, as we have commented above, BIR is associated with the activation of tau kinases, including GSK3β, through the phosphatidylinositol kinase (PI3K)/protein kinase B (AKT) signaling pathway [60]. Thus, BIR leads to an overactivation of GSK3β, which in turn promote tau hyperphosphorylation. Likewise, tau hyperphosphorylation, oligomerization, misfolding, and aggregation are involved in the impairment of synaptic plasticity and contribute to the neurodegenerative process due to its location on dendrites in the postsynaptic terminals [61]. It has been reported that deposits of p-tau in the CA1 and CA3 regions of hippocampus are related with the decrease of density and shape of dendritic spines, as well as neuronal loss [60,61]. Yarchoan and colleagues reported that BIR was related to IRS1-pS616 and IRS1-pS312 expression in LOAD and brain tauopathies, including Pick’s disease, corticobasal degeneration, and progressive supranuclear palsy [61]. Thus, in this study, the authors suggest an association between BIR and tau, in which IRS-1 pS616 phosphorylation increases favor an abnormal tau phosphorylation [61].

In this line, Marciniak and colleagues demonstrated the role of tau as a key modulator of BIR and the insulin receptor signaling pathway, as well as the mechanisms whereby tau could modulate insulin receptor function [59]. Hence, tau deletion is involved in the control of peripheral and brain insulin metabolism, the modulation of hippocampal BIR can contribute to cognitive function and hypothalamic BIR regulates metabolic alterations in LOAD patients and in tauopathies [59]. Therefore, chronic BIR is involved in the development of tau pathology by altering the balance between kinases and phosphatases and vice versa. Moreover, it has been demonstrated that tau hyperphosphorylation leads to an increase in uptake and intraneuronal accumulation of insulin as insoluble oligomeric aggregates in LOAD patients and in several tauopathies [58]. Interestingly, this process occurs independently of T2DM, suggesting that BIR is associated with alterations of insulin signaling pathway independently of the presence of clinical T2DM.

Likewise, tau has recently been identified as a key regulator of peripheral insulin signaling, with evidence linking tau to IR in the brain and peripheral tissues, as well as beta cell dysfunction [60,62,63]. Tau is widely expressed in insulin-secreting beta cells in the pancreatic islets. At a young age, mice with a global tau knockout exhibit a rise in body weight, defects in glucose-stimulated insulin secretion, and reduced glucose tolerance [60,61].

Although it is well known that insulin can modulate the phosphorylation of tau protein, its role in the regulation of the insulin receptor has been only studied in the recent years. In accordance with this idea, mice which do not express insulin receptors at the neuronal level (NIRKO mice) as well as IRS-2^-/-^ mice showed an increased phosphorylation of tau through the inhibition of phosphoinositide 3 kinase (PI3K)/AKT signaling pathway [18,19,62,63]. This link between insulin and tau signaling is mainly based on the modulation of downstream signaling pathways involving different kinases such as GSK-3β, c-Jun N-terminal kinase (JNK) and AMPK and phosphatases including protein phosphatase 1 and 2 (PP1 and PP2A, respectively) [18,19].

## 3. Metformin as an Antidiabetic Drug Strategy for Alzheimer’s Disease Treatment

Metformin is an antidiabetic drug derived from galengine, a natural product of the *Galega officinalis* plant [18]. Metformin is a biguanide that contains two couple guanidine molecules [64,65,66], with a highly hydrophilic chemical structure (1, 1-dimethylbiguanide hydrochloride) properties [65]. Therapeutically, metformin is the first-line treatment for T2DM and is prescribed by most health guidelines because its low side-effects, it is usually well absorbed, and not associated with weight gain [67]. Metformin decreases liver gluconeogenesis and reduces insulin resistance, leading to lower levels of plasma glucose [65]. Likewise, metformin is able to cross the blood–brain barrier (BBB) and has been involved in increased cognitive performance [68]. Furthermore, metformin could alter gut microbiota composition, which may play a role in AD pathogenesis [69].

### 3.1. Preclinical Animal Studies with Metformin

For the purpose of designing therapeutics or disease modifying agents for AD treatment, a wide variety of animal models have been developed to replicate the human environment of the disease [70,71,72,73,74]. In particular, the first aim of most AD animal models is to develop the neuropathological features that precede the cognitive dysfunction [75,76,77].

The transgenic mice are important models for deciphering familial AD pathology pathways. These models do not display all the anomalies found in human AD and do not duplicate the sporadic forms of AD, because they only reproduce the pathological features of AD common mutated genes [77]. However, transgenic innovation gives special opportunity to replicate the cause of familial AD by transfecting a mutant human APP [74,75]. Mice models enabled our understanding of Aβ-production, deposition, and clearance-related molecular pathways and the impact of Aβ on the neuronal network and synapses [73,74]. A wide variety of parenchymal and vascular amyloid deposits similar to those of human AD were developed successfully by the APP mouse model [75]. Transgenic mice models developed by over-expression of mutated human PS1, APP and tau, are the majority of these animal models. The triple-transgenic 3xTg-AD mice contains three mutated genes (human PS1M146V, APPSwe and tauP301L) and develops increasing age-dependent amyloid plaques and NFTs as well as memory deficits [76,77,78] (Table 1).

On the other hand, many of the signature features of AD are reproduced by the injection of pharmacological or chemical agents into the brain or by the activation of lesions in specific brain regions [76,77,78]. For instance, the injection of Aβ peptide into the brain of rat or rhesus monkey has been used in several studies. Although these models cause some of the clinical signs, they do not specifically mimic AD pathology. Lesion models include the chemical or physical degradation of particular regions of the brain such as hippocampal, cortical, and striatal regions that are normally either cholinergic or active in cognitive processes [1]. In general, interventional models will be effective for detecting symptomatic or therapeutic interventions as a disease model. These models can include valuable observations such as the streptozotocin (STZ)-induced AD model, scopolamine-mediated amnesia model that led to learning and memory loss and cognition dysfunction [76,77,78,79,80]. For instance, in scopolamine-induced amnesia models, inflammation is activated by endotoxins and the brain metabolism interacts with other chemical action models [73].

Since LOAD represents more than 95% of AD cases, associated animal models are valuable research resources for studying pathogenesis and designing experimental treatments for sporadic AD [76,77,78,79,80,81,82,83,84]. STZ is a diabetogenic agent that is widely used to induce diabetes in animals because it damages and induces IR in pancreatic beta cells. Decrease of glucose/energy metabolism in brain, corresponds to the severity of dementia symptoms in AD, and is a well-established brain abnormality of sporadic AD [77,78,79,80,81,82,83,84,85,86,87,88,89,90,91,92,93]. In ICV-STZ animal models, BIR reduced brain glucose metabolism, tau and Aβ accumulation, gliosis, cholinergic deficits, oxidative stress, and learning and memory deficits [77,78,79,80,81,82,83,84,85]. Brain insulin signaling controls the metabolism of cerebral glucose, and impaired transduction of brain insulin signaling was reported in AD [82]. Tau hyperphosphorylation, increasing of Aβ40/42 and both GSK-3β and BACE1 activities have also been observed in rats treated with STZ, which also exhibited a lack of dendritic and synaptic plasticity [85,86,87]. In January 2013, Dr Hoyer hypothesized that ICV STZ is the non-transgenic metabolic form of sporadic AD [85]. Hoyer’s reasoning began with the observation that while both oxygen and glucose intake in the brain decreases in LOAD, the decrease in brain oxygen consumption is significantly smaller [85,86,87,88,89]. These findings lead to the hypothesis that the main biochemical change in incipient LOAD is related to the regulation of cerebral glucose metabolism, which leads to an alteration of a signal transduction deficiency of the cerebral insulin receptor [89]. According to findings of Saffari and colleagues, an i.c.v. injection of STZ caused a substantial decrease in spatial learning and memory, while metformin administered in therapy phosphatidylserine nanoliposomes formulation improved learning and memory. Thus, metformin increased spatial learning and decrease neuroinflammation in the STZ rat model of LOAD [89]. In addition, the recent results of Pilipenko et al. showed that metformin reversed STZ-induced impairments in spatial learning/memory capacity and sociability, as well as normalization of brain glucose transport, uptake, and metabolism, together with an improved microgliosis and astrogliosis in a LOAD rat model [84].

According to the studies of Ditacchio and colleagues, metformin is an efficient treatment to improve insulin sensitivity, with a higher drop in blood glucose levels in the AβPP AD model [90]. In addition, Farr and colleagues examined the effect of metformin on the expression of APPc99, AβPP, Aβ, G3DPH, and p-tau in SAMP8 mice [91]. They showed that the expression of APPc99 and p-tau decreased after metformin treatment. Thus, metformin treatment in SAMP8 significantly reduced hyperphosphorelated tau and APPc99 proteins, leading to an improve in learning and memory processes. Moreover, Ditacchio and colleagues also showed that Aβpp transgenic female mice that were treated with metformin showed increased cognitive abilities [90] (Table 1). These results were seen in another genetic model of AMPK activation, where some unexplained structural mechanism disrupted AD-related cognitive activity in these animals downstream of liver AMPK activation [91]. Additionally, the authors carried out studies in males and females and the results demonstrated that beneficial effects of metformin were greater in females than in males [91]. This supports the idea that there may be an effect of the gender in the effectivity of this drug.

Finally, Lu and colleagues demonstrated that metformin improved learning and memory performance in APP/PS1 transgenic mice, according to Morris water maze and Y-maze results [93]. Another study demonstrated that metformin improved microglial autophagy in the APP/PS1 mice model, allowing pathological Aβ and tau proteins to be phagocytized, and thus reducing Aβ deposits and restricted the distribution of tau pathology [83]. Similarly, Ou and colleagues also reported that metformin treatment in APP/PS1 exerts multiple beneficial effects in the brain neuropathology [82]. Thus, metformin treatment improved the cognitive process and neurogenesis, exerting neuroprotective effects on the hippocampus. Moreover, metformin, probably through the modulation of the AMPK/mTOR/S6K/BACE1 signaling pathway, also improved amyloidogenic pathway and prevented the neuroinflammatory process [82].

**Table 1:** Effect of different dose of metformin on treatment of preclinical Alzheimer’s disease.

Row	Reference	Animal Model/Gender	Starting Age	Metformin Dose	Duration of Therapy	Main Finding
1	[82]	B6C3-Tg (APPswe, PS1dE9) 85Dbo-fAD/F	26 weeks old	200 mg/kg/d	14 days	Neuroprotection, Enhanced memory, reduced inflammation, regulation of AMPK/mTOR/S6K/Bace1 pathway.
2	[91]	SAMP8 mouse model of random onset- AD/M	12 months old	20–200 mg/kg/d	8 weeks	Increased PKC, improved pGSK-3ser9,reduced pTau404 and APPc99, enhanced learning and memory.
3	[90]	PDAPP (J9) mice-AD/M&F	6–8 weeks	350 mg/kg/d	Until 14–16 months-old	increases insulin sensitivity in male, lifespan extension and delayed degradation of the estrous cycle in female
4	[94]	C57BL/6 mice-PD/M	10-weeks	200 mg/kg/d	10 days.	Stimulate AMPK, mediating the pleiotropy
5	[95]	Wistar rats-AD/M	Five-month old	50, 100–200 mg/kg/d	3 weeks	Decreasing Memory loss, preserved the pAMPK and CREB levels, Improved TAS & SOD levels, increased antioxidant function
6	[96]	Wistar rats-AD/M	Adult	100 mg/kg/d	8 weeks	Enhances neuronal activity and neuropathological modifications, prevent synaptic plasticity impairment
7	[83]	Wistar rats-sAD/M	9 weeks	75–100 mg/kg/d	21 days	Modulation of glucose delivery and uptake, anti-neuroinflammatory function, maintenance of synaptic plasticity
8	[86]	C57BL/6 mice-sAD/M	12–14 weeks	200 mg/kg/d	21 days	Suppress glycemic levels and cognitive dysfunction, increases insulin receptor sensitivity, facilitate neuronal survival
9	[76]	APP/PS1 transgenic mice/F	9 months old	4 mg/mL in drinking water	2 months	Promoted the phagocytosis of Aβ and tau proteins by enhancing microglial autophagy capability

### 3.2. Metformin in Clinical Studies

It has been shown that metformin stops or slows the onset of dementia in adults with diabetes [97]. In 2019, Shi and colleagues focused on the effect of metformin in elderly adult US veterans with T2DM and neurodegeneration [98]. According to the results of this study, metformin therapy over 2–4 years provides a strong risk reduction in the occurrence of neurodegeneration in patients with T2DM compared with patients without metformin treatment [98].

A pilot study of 80 people with amnestic moderate cognitive disorder was undertaken at Columbia University in New York City from 2008 to 2012. The participants were overweight, but none of them had diabetes. They were given either 2000 mg of metformin separated into two doses or a placebo for one year. The selective reminding test (SRT) for recall and the ADAS-Cog were the primary outcomes [99]. The secondary endpoint was FDG-PET glucose absorption in the posterior cingulate/precuneus, as well as plasma levels of Aβ42, the most toxic form of the Aβ peptide. The metformin group performed slightly higher on the SRT than the placebo group. There were no differences in the ADAS-Cog, glucose uptake, or plasma Aβ42 between classes. Only 10% of patients were able to take the peak dosage of metformin, with the majority receiving 1000 or 1500 mg a day [92,93]. The main conclusion of the study was that metformin improve of efficacy for recall in the SRT.

From 2013 to 2015 a small study at the University of Pennsylvania assessed the impact of metformin on biomarkers of AD in 20 non-diabetic individuals with moderate cognitive dysfunction or dementia related to the disease. MRI, FDG-PET, and amyloid biomarkers were used to validate the diagnosis of AD [100,101]. Each participant was given metformin at a daily dose of 2000 mg/day for eight weeks, then placebo for eight weeks, or vice versa, in a crossover study. The ADAS-Cog and CANTAB batteries were used to assess cognitive performance in multiple learning and memory domains, executive processing, focus, expression, and motor speed. Cerebral spine fluid (CSF) concentrations of Aβ, total tau, and tau were also evaluated, and blood flow in the brain was determined by arterial spin marking [9]. In the treated population, the Trails B test of executive function showed a statistically significant increase, as well as improvements in learning, memory, and focus. Metformin had little effect on blood supply in the areas where it was tested. The compound was found in the CSF, but the AD biomarkers remained unchanged [101].

In February 2020, Swedish researchers began testing the impact of a year of metformin treatment plus exercise and diet on memory in 80 people with T2DM and moderate cognitive dysfunction. Recruitment, adherence, and retention rates are the primary consequences, while metabolic improvement and memory capacity are secondary measures. The research will last until December 2021 (https://www.alzforum.org/therapeutics/metformin) (accessed on 23 August 2021).

In turn, Samaras and colleagues compared the efficacy of metformin on cognitive decline and dementia risk in diabetic patients. After 6 years of research, authors concluded that the administration of metformin in older people with T2DM was associated with a decreased risk of dementia [102]. In addition, in an interesting study performed in aged African American and white patients, with data taken from Veterans Health Administration (VHA) medical record, Scherrer and colleagues showed that the administration of metformin decreases the risk of dementia by 29% and 40% in African American patients aged 65 to 74 years and 50 to 64 years, respectively. These results are very interesting because they give support to the hypothesis that metformin is able to decrease the risk of dementia in aged patients [103].

On another front, Sluggett and colleagues demonstrated that Finnish patients with T2DM and long-term metformin treatment had lower risk of developing AD. Again, the results of this study give support to the hypothesis that glucose lowering drugs may be important pharmacological alternatives that modify the course of the disease and delays the risk of dementia [104].

On the contrary, other studies such as those of Koo and colleagues showed that metformin treatment was not effective and even worsened the cognitive state in older Korean patients [105]. For this reason, more studies are necessary to clarify metformin effects in diabetic patients with cognitive loss [106,107].

## 4. Molecular Mechanism Involved in Neuroprotective Effects of Metformin in Alzheimer’s Disease

### 4.1. Metformin Effects on Amyloid and Tau

Previous studies have reported that AMPK is highly expressed in the hippocampus —a brain region that plays key roles in synaptic plasticity, memory, and cognition—and aberrant AMPK activity has been reported in the brains of transgenic mouse models of AD and AD patients [108,109]. Based in the amyloidogenic hypothesis of AD, it has been reported that Aβ oligomers inhibited AMPK and thus could increase the risk of a metabolic dysfunction in hippocampal neurons that may play a key role in early metabolic defects in the LOAD brain [110]. Thus, metformin, which is able to promote AMPK activation, could be an attractive target that can compensate this energy loss in the in the nervous system. In addition, AMPK activation can reduce Aβ by reducing BACE1 expression and thus decrease brain Aβ levels [111,112]. Moreover, AMPK could play an additional favorable role in LOAD by promoting autophagy [70,71,72]. Indeed, previous studies have reported that activation of autophagy decreased Aβ pathology and improved the cognitive process in preclinical models [113]. Therefore, metformin could improve the brain autophagic function, helping to remove waste proteins and improving the treatment of AD [113].

It is well known that phosphorylation of tau is regulated by several kinases, including AMPK, which is a tau kinase that acts by phosphorylating multiple tau sites [114,115]. However, the process of tau regulation by AMPK is complex since it can be regulated by direct and indirect mechanisms. Wang and colleagues reported that both salicylate, an AMPK agonist, and wortmannin, a GSK-3β inhibitor, reduce tau phosphorylation [61]. Likewise, AMPK can phosphorylate the Ser9 site of GSK-3β, triggering its inhibition, and therefore it may explain its participation in the modulation of this regulatory process in the phosphorylation of tau [116,117]. Apart from the direct regulation of tau phosphorylation, AMPK also activates SIRT1, a deacetylase enzyme which, by improving or enhancing the deacetylation process, can inhibit the hyperphosphorylation of tau [115]. Likewise, another mechanism that regulates both the acetylation and phosphorylation of tau involves the protein phosphatase 2A (PP2A). Interestingly, it was reported that metformin induces tau dephosphorylation by directly activating PP2A [117]. In addition, PP2A activity is increased by AMPK-mediated phosphorylation at Ser298 and Ser336 [98,99].

In general, the role of metformin on AMPK activation and phosphorylation of tau is not fully understood, since involves both direct and indirect mechanisms. Because different hypotheses have been proposed, more research studies are needed. However, it is accepted that metformin improves mitochondrial defects, promotes the autophagy, and regulates insulin sensitization through the modulation of different intracellular pathways and consequently could improve LOAD neuropathology in preclinical AD models [104,105,106,107]. Therefore, metformin could be a suitable potential therapeutic treatment of metabolic risk factors target for LOAD.

### 4.2. Metformin Effects on Mitochondria

A strategy based on “brain energy rescue” in the treatment of LOAD has currently been proposed [118]. The objective of this strategy is based on preserving and/or restoring the energy state of the brain. In this sense, metformin treatment could be a potential brain energy rescue strategy by improving mitochondrial function and improving peripheral and cerebral glucose metabolism. It is well known that mitochondrial metabolic abnormalities are involved in the pathogenesis of LOAD [114,115]. Thereby, it has been proposed that AMPK can regulate mitochondrial synthesis and the main functions of mitochondrial autophagy. Previous studies have shown that mitochondrial damage is an early sign of LOAD that appears before NFTs and is accompanied by phosphorylation of the tau protein [115]. Thus, the activation of AMPK through metformin can favor the process of mitochondrial biogenesis regulating the function of peroxisome proliferator activated receptor γ coactivator-1α and peroxisome coactivator-1α (PGC-1α, a transcriptional coactivator nuclear) [110]. Furthermore, as we have already commented above, metformin could promote the mitochondrial autophagy process through AMPK activation, hence favoring the elimination of damaged/defective mitochondria, increasing ATP production, and reducing the production of reactive oxygen species [116]. Therefore, it can be hypothesized that the activation of AMPK by metformin could generate an increase in cellular autophagy and ATP production and helps to improve the symptoms of LOAD.

### 4.3. Metformin Effects on Neurogenesis: The AMPK/aPKC/CBP Signaling Pathway

Metformin is involved in two distinct molecular pathways to facilitate the proliferation/regeneration and differentiation of adult neuron progenitor cells (NPCs) [119]. In the first pathway, metformin activates AMPK, which activates the cascade of aPKC-CBP to facilitate neuronal differentiation. Atypical protein kinase C (aPKC) is stimulated upon activation of AMPK, which ultimately phosphorylates CREB-binding protein (CBP) at Ser133 to facilitate neurogenesis and increase spatial memory development in adult mice [119,120]. In the second pathway, metformin significantly upregulates the expression of TAp73 mRNA, which in turn increases the production of essential proteins involving in self-renewal of adult NPCs. P73 is a transcription factor that plays a key role in neural stem cells and its expression increases following their differentiation [120]. For the treatment of patients with cognitive dysfunction associated with T1DM and T2DM, the ability of metformin to stimulate neurogenesis is potentially promising [117,118,120]. According to the previous studies, it can be shown that long-term usage of oral metformin therapy improves hippocampal neurogenesis and spatial memory, followed by an induction of chronic microglial activation and improved glucose-lowering impact of phosphorus-relation of AMPK/aPKC f/k/IRS1 serine residues in the hippocampus of middle-aged diabetic mice [120]. These findings are consistent with previous research demonstrating the neuroprotective effects of chronic metformin administration on high fat diet-induced deterioration in hippocampal neurogenesis and neurological disorders [117]. Taken together, considering the crucial roles of AMPK in intracellular metabolism in LOAD, metformin could be introduced as suitable and attractive therapeutic target [116].

Studies by Ma and colleagues showed that metformin improves the composition of the gut microbiota of obese mice. This peripheral effect could inhibit the neuroinflammatory process in the hippocampus. Likewise, this drug could prevent the deterioration of newborn neurons in the hippocampus and therefore improve the learning process and memory in obese mice. These results reinforce the hypothesis of the benefit of acting at the microbiota level to improve the cognitive process in LOAD [121].

### 4.4. Metformin Effects on Learning and Memory

Cognition is one of the most complex features of the brain, and it involves perception, registration, consolidation, storage, and memory over the course of human life [118]. Any memory deficiency, such as amnesia, has a significant impact on an individual’s quality of life and is regarded as a major CNS disease attributed to a decline in neuronal population as a result of ageing, neurodegenerative disorders, head injuries, brain defects, genetic anomalies, and other factors [122]. Accumulating evidence suggests that diabetic therapies in model animals or humans with diabetes can improve cognitive functions. Likewise, thiazolidine-based diabetic therapy, i.e., pioglitazone, decreases the risk of dementia in patients with diabetes and increases both glucose metabolism and memory performance in patients with LOAD and diabetes [122,123]. Treatment with metformin has shown to substantially enhance memory deficits. Mostafa and colleague’s studied the acute administration of metformin in a scopolamine-amnesic mice model (with impaired learning and memory skills), for about two weeks, and demonstrated the valuable effects of metformin on improving memory [71]. The molecular mechanism involved in the neuroprotective action of metformin was multiple since it showed significant antioxidant and anti-inflammatory activity. However, the authors propose that its protective effect against scopolamine-induced cognitive impairment is probably through the signaling pathway of Akt/GSK3 beta and prevention of phosphorylation of tau protein. Likewise, according to some trials, scopolamine therapy has been shown to decrease pAMPK and CREB levels, and metformin treatment has successfully restored pAMPK and the transcription factor CREB levels in the hippocampus [71,121]. Furthermore, metformin was able to increase the hippocampal levels of antioxidant enzymes, such as superoxide dismutase levels [121]. These results give support to the hypothesis that metformin could be a potential preventive drug against cognitive and memory impairment [94,95,121,124,125,126,127,128,129,130,131,132] (Table 1). Similarly, metformin was shown to prevent cognitive damage in the chronic L-methionine model of memory impairment, probably by normalizing oxidative damage [124].

Kodali and colleagues reported that after 10 weeks of metformin treatment, C57BL6/J mice with late middle age improved recognition memories in old age [72]. Metformin treatment in the hippocampus modulated microglial cells in an anti-inflammatory M2 phenotype and reduced hypertrophy of astrocytes. Furthermore, it reduced the concentration of proinflammatory cytokines and enhanced autophagy processes through the activation of AMPK and inhibition of mTOR signaling.

Likewise, since the hippocampus is an essential part of the brain for memory and cognition and it is widely affected in AD, the hippocampus’ neuronal activation and synaptic transmission are important for improving these functions. According to Chen and colleagues, metformin improved synapsis, memory, and cognitive deficits with disrupted hippocampal synaptic communication [131]. This process could be explained through the increased presynaptic glutamate release, which would be responsible for the increased elevated miniature excitatory postsynaptic currents (mEPSC) into CA1 pyramid neurons in hippocampus [131]. Likewise, Asadbegi and colleagues demonstrated that metformin treatment was able to improve significantly long-term potentiation in rats after the Aβ-injection. Moreover, rats were under a high-fat diet (HFD), and metformin treatment showed neuroprotective effects against detrimental effects of Aβ and HFD on hippocampal synaptic plasticity [96].

In turn, Li and colleagues studied the effects of intraperitoneal injection of 200 mg kg^−1^ d^−1^ of metformin for 18 weeks in db/db mice, which have multiple AD-like brain changes such as alterations in cognitive functions, increased phospho-tau and Aβ, as well as decreased synaptic proteins. Metformin decreased hippocampal levels of total tau, phospho-tau, and activated c-Jun-N-terminal kinase [133]. Moreover, metformin treatment increased the levels of synaptophysin, a synaptic protein, in the hippocampus of db/db mice [133]. Notwithstanding, metformin did not attenuate spatial learning and memory deficits. However, it was effective in enhancing biochemical changes like those of AD in the hippocampus of these mice.

### 4.5. Metformin Effects on Synaptic Density and Dendritic Spines

The theory that activating insulin receptors enhances cognition has been confirmed by clinical and preclinical trials. Hence, it is accepted that insulin improves emotional function in active and elderly people, as well as Alzheimer’s patients [128]. Insulin signaling can influence the synaptic plasticity by controlling glutamate receptor expression and trafficking, and insulin receptors are enriched at hippocampal synapses, where they are proposed to control synaptic plasticity by interactions with the glutamatergic system [95,128,129,130,131]. Furthermore, various studies have shown that synaptic markers and/or dendritic spine dysfunction appear before the development of Aβ plaques and NFTs, thus implying that these events are closely linked to cognitive decline in AD [95,128,129,130,131,132]. In older rhesus monkeys, selective loss of thin spines is closely associated with decreased learning capacity [134,135,136]. In addition, according to Morrison and Baxter, reducing spine form can have a detrimental impact on prefrontal synaptic plasticity, which is essential for normal functioning in aged people [137]. In this line, the maintenance of thin and mushroom spine populations (another spine type) combined with cumulative increased spine extent in the dorsal-lateral-prefrontal-cortex (DLPFC) distinguish cognitively normal older individuals with AD pathology from patients with AD dementia [134]. This changes may be linked to the mild cognitive impairment (MCI) that can be detected early in AD patients [134,135,136], confirming that synaptic loss is key to the development of the disease [60] and supplying cellular evidence that dendritic spine remodeling could be a process of cognitive resilience. All these findings support the idea that synaptic function and behavior are directly related to cognition ability [138,139,140,141]. As a result, the cellular and molecular events that regulate synapses may be used to treat cognitive dysfunction in AD [136].

Loss of synaptic activity in the AD brain can be correlated with observed cognitive deficiencies [12,138,139,140,141]. Metformin has been shown to mediate memory forming through synapse plasticity [142,143]. In addition to synaptic impairment and lack of neuronal integrity in mature neuronal circuitry, in the AD-associated neurodegenerative phase, aberrant adult hippocampal neurogenesis is also involved. Cyclin-dependent kinase 5 (CDK5) is a serine/threonine kinase triggered by p35/p39 neuron-specific activators that plays a key role in synaptic plasticity neuronal and cognitive behavior [144]. The proteolytic cleavage of p35 to p25 contributes to protracted and aberrant activation of CDK5 and results in synaptic depression, which closely mimics early AD pathology [145]. Consequently, a possible promising strategy for the development of AD drugs is CDK5 inhibition. It has been found that metformin inhibited CDK5 hyper-activation and CDK5-dependent tau hyper-phosphorylation in the hippocampus of APP/PS1 mice [144,145,146]. Liu and colleagues reported that CDK5-activation by hyperglycemia is involved in neuronal apoptosis [145]. Furthermore, it was shown that CDK5 phosphorylates the PPARγ receptor on serine residue 273, thus preventing the transcription of antiobesity effects and favoring weight gain. In this sense, Cai and colleagues reported that CDK5 could be the link between AD and T2DM hyperacetylation of H3K9 histone on CDK5 promoter [146].

The transgenic APP/PS1 mice presents loss of spines as demonstrated by reduced spine density from CA1 pyramidal neurons. According to Wang and colleagues’ study, chronic metformin administration for 10 days improves synaptic defects, including surface GluA1 expression, decrease spine disappearance, and reduction in basal synaptic transmission in the hippocampus of APP/PS1 mice [144]. Furthermore, in highly primed hippocampal slices from APP/PS1 mice, theta burst stimulation-induced CA3-CA1 long term potentiation (LTP) was compromised, while the LTP deficiency was saved by chronic therapy with metformin for 10 days too [144]. Increased presynaptic glutamate release from terminals innervating CA1 hippocampal pyramidal neurons was observed using paired-pulse ratios (PPR), but the excitability of CA1 pyramidal neurons was not affected. These findings indicate that metformin improves glutamatergic rather than GABAergic signaling in hippocampal CA1, revealing new information about metformin’s actions on neurons.

### 4.6. Metformin Effects on Neuroinflammation

According to Ha and colleagues, metformin possesses anti-inflammatory effects [147]. The neuroinflammatory response requires microglial cells, which are resident phagocytes in the CNS. Microglial cells initiate an innate immune response when they are triggered by danger-associated molecular patterns (DAMPs) such as S100A8, S100A9, Aβ, or pathogen-associated molecular patterns (PAMPs) such as lipopolysaccharides (LPS) [147]. According to reduction of multiple inflammatory responses in BV-2 microglial cells by metformin treatment, including the secretion of pro-inflammatory cytokines such as tumor necrosis factor(TNF)-α and interleukin (IL)-6, it could be considered an important autophagy regulator and anti-neuroinflammatory drug [147]. Pursuant to Liu and colleagues, metformin reduced the incidence of clinical stroke in adults with diabetes and attenuated post-stroke brain atrophy volume 24 h after therapy in mice with temporary middle cerebral artery occlusion (tMCAO). In normal mice, metformin therapy not only stimulated neurogenesis through the modulation of the CREB-binding protein (CBP)-protein kinase C (PKC) pathway, but also increased lifespan by alleviating chronic inflammation [148]. Accumulation of Aβ increases the proinflammatory factors IL-1β and IL-6 levels in APP/PS1 mice [76]. It has been reported that metformin decreases the levels of IL-1β and IL-6 in APP/PS1 mice [76]. In addition, different studies have highlighted the anti-inflammatory and antioxidant function of metformin, with several pathways playing a key role in the activation of AMPK [147,148,149,150,151,152]. In certain cases, metformin suppresses inflammation and decreases or removes inflammatory factors largely by dependent pathways and often independently of AMPK at the cellular level and elsewhere at the systemic level [148,149]. Metformin is also efficient in decreasing the amount of oxidative stress factors by controlling the cell’s antioxidant function [64,65]. Interestingly, metformin anti-inflammatory effects could be due to the decrease of the expression of nuclear factor kappa B (NF-κB) [65]. NF-ⱪB is involved in multiple inflammatory pathways, cell death, and tissue degradation [147]. Moreover, it is widely demonstrated that AGEs are one of the most important inflammatory factors in the development process of diabetes. Macrophages actively participate in this inflammatory process, which act by amplifying the expression of pro-inflammatory cytokines (IL-1, IL-6, and TNF-α), in addition to increasing the expression of receptor for RAGE and activating the NF-κβB pathway. Indeed, RAGE/NF-kB signaling plays a role in the inflammatory activity of AGE-stimulated macrophages/microglial cells. By activating AMPK and inhibiting NF-ⱪβ, metformin suppresses the RAGE/NF-kβ pathway, which leads to inhibited effects of AGE and at the brain level it can decrease the activation of microglia favoring the M2 (anti-inflammatory) phenotype over M1 (classic or inflammatory) [149].

On another front, it has been reported that metformin can decrease ROS production through direct inhibition of the chain of the electron transfer complex of complex I (NADH ubiquitin oxidoreductase (NADH) [151,152,153]. Other described mechanisms involved in the reduction of ROS may be due to the activation of antioxidant enzymes such as catalase, which is the main decomposer of H_2_O_2_, inducing the endogenous antioxidant system that includes glutathione reductase (GSH), superoxide dismutase (SOD), and catalase (CAT [151,152]. It has also been described that metformin can stabilize the nuclear factor related to erythroid 2 (Nrf2), a sensor of oxidative stress, and induce its gene expression, through AMPK. Induction of the Nrf2 pathway is associated with an increased level of antioxidant system enzymes such as CAT, GSH, and SOD [151,152,153]. Thus, through the induction of AMPK activation, metformin stimulates the initiation of this pathway and may explain its antioxidant function.

In several studies performed on mice with traumatic spinal cord injury, reactions of local inflammation along with microglia proliferation, activation, and phagocyte infiltration are used [154]. In a demyelinating context induced by lysolecithin, metformin treatment reduced demyelination and inflammation and protected the functional integrity of optic tract, as measured by visual evoked potential recording [155]. Moreover, potential application of metformin in multiple sclerosis has been recently reviewed [154].

All these data support that metformin has an antioxidant and anti-inflammatory function in different circumstances. Therefore we can conclude that, for several neurodegenerative diseases whose inflammatory pathways and oxidative stress play a role in their pathogenesis, metformin may be an effective therapeutic choice [150,151,152].

### 4.7. Neuroprotective and Neurorestorative Potential of Metformin

Chung and colleagues studied the genes and proteins whose expressions or functions were either directly or indirectly influenced by the AMPK pathway to understand the role of metformin between multiple signal pathways in neuroprotection [124]. AMPK can work independently via various roles of many basic cell type functions (e.g., mitochondrial biogenesis, cellular synthetic activity, anti-inflammation, anti-oxidative stress, cell growth, and proliferation) and molecular pathways (e.g., incorporation of proper effects through AMPK-PPARγ, AMPK-PGC1 alpha, AMPK-PFK, AMPK-FOXO, and AMPK-mTOR signaling cascades) [124]. Downregulation of AMPK and downstream signaling pathways, lead to AGEs production, to an increase in human neural stem cells (hNSC) death and to mitochondrial dysfunction. Several studies have also shown that AGEs reduce mitochondrial capacity, and Wareski and colleagues demonstrated that AMPK stimulation promotes mitochondrial activity through the activation of PGC1α [153]. In age treated hNSCs, metformin also improves AMPK, PGC1α, NRF-1, and Tfam expressions that may contribute to the observed elevation in mitochondrial functions. In addition, metformin-enhanced neuroprotective gene expression can help to protect hNSCs against toxicity caused by AGE [150].

Interestingly, Fatt and colleagues demonstrated a potential neurorestorative effect of metformin [153]. They reported that treatment with metformin improves NPCs proliferation, self-renewal, and neuronal differentiation [153]. Metformin therapy orchestrated this process mainly through the activation of TAp73 gene expression in adult NPCs and through AMPK activation by triggering the cascade of aPKC-CBP [153].

## 5. Conclusions

Dementia is linked to a number of co-morbid disorders in the elderly, including diabetes, asthma, dyslipidemia and cardiovascular disease, among others [1,4,6]. Therefore, all these factors could substantially complicate LOAD treatment. For this reason, it has been proposed that a combination therapy with more than one drug may be necessary to slow or delay the evolution of the disease [2,3,4,5]. In this regard, in a combinatory therapy with 3 or 4 drugs (anticholinergics, memantine, aducanumab, sodium oligomannate (GV-971), anti-inflammatories) it may be interesting to add a drug such as metformin, which may be key in improving hypometabolism and increasing glucose uptake in the brain. For this reason, metformin (or other antidiabetic drugs) can provide added value by increasing glucose transport to neurons and increasing ATP levels [65,149,150,151,152,153]. Thus, based on literature, metformin can be used to inhibit dementia progression and can be a novel therapeutic medication for strengthening LOAD-related cognitive dysfunction [65].

In general, metformin is considered a safe and well-tolerated drug. However, the appearance of gastrointestinal adverse effects—such as diarrhea, nausea, and vomiting—has been reported [154]. Less frequent may be the appearance of headache, hypoglycemia, weakness, and rhinitis. However, one must be very careful as metformin has a serious warning for the risk of lactic acidosis [156]. This side effect is rare but serious and has an incidence rate of 1 in 30,000 patients. In this way, metabolic acidosis results in a decrease in the pH in the blood causing nonspecific signs and symptoms, such as respiratory distress, elevated lactate levels and acidosis [156]. Lactic acidosis can, in turn, cause hypotension, hypothermia, and death.

**Figure 1:**
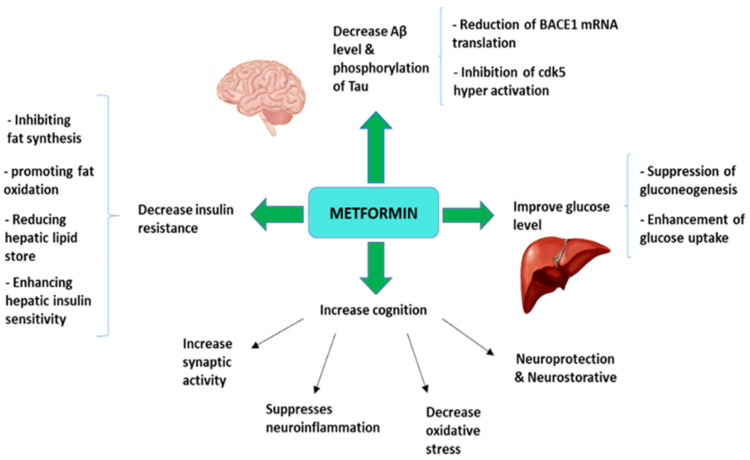
Metformin decreases the insulin resistance by multiple mechanisms and increases insulin sensitivity [18]. Reduction in level of Aβ and phosphorylation of Tau protein by reduction of BACE1 translation [67] and improving the glucose level by suppression of gluconeogenesis are the other beneficial effects of metformin treatment in neurodegenerative disorders such as AD [18]. Metformin also improves cognition by increasing synaptic activity [64,65,131], suppressing inflammation and decreasing oxidative stress [75]. In addition, metformin has neuroprotection and neurorestorative effects to increase memory, learning, and cognition in AD cases [101].

Metformin, through multi-directional pathways, could be a promising candidate for prevention of not only LOAD, but also of other neurodegenerative diseases, due to beneficial effects at the central and peripheral level (Figure 1). Metformin crosses the BBB and acts centrally via exerting a neuroprotective effect. It may also facilitate neurogenesis and enhance spatial memory development. In addition to cognitive and behavioral changes that follow the emergence of LOAD, recent findings indicate that metformin could play a neuroprotective role by correcting the hallmarks of brain damage (metabolic dysfunction, synaptic dystrophy and cellular loss). Preclinical results of metformin treatment on transgenic mice, demonstrate that spatial memory can be improved as well as neuroprotection and neurogenesis in hippocampus. In addition, amyloidogenesis and inflammatory reactions can be affected by metformin to decreasing through regulation of AMPK/mTOR/S6K/Bace1 signaling and block the NF-kβ. Regarding clinical trials, the authors generally suggest that future studies should include biomarkers of AD in CSF or image markers such as PET associated with amyloid ligands, so that the results reinforce the modifying role of metformin in the LOAD. In turn, studies with metformin in older people with diabetes showed that this drug was associated with an improvement in global cognition and reduced the risk of dementia compared to older people with diabetes who were not treated with metformin. Therefore, we must wait for the results of more clinical studies to confirm the role of metformin in a potential combinatorial therapy in the prevention of LOAD.

## Data Availability

No new data were created or analyzed in this study. Data sharing is not applicable to this article.

## References

[1] Scheltens P., De Strooper B., Kivipelto M., Holstege H., Chételat G., Teunissen C.E., Cummings J., van der Flier W.M. (2021). Alzheimer’s disease. Lancet.

[2] Alzheimer’s Association (2021). 2021 Alzheimer’s disease facts and figures. Alzheimers Dement..

[3] Cummings J., Lee G., Zhong K., Fonseca J., Taghva K. (2021). Alzheimer’s disease drug development pipeline: 2021. Alzheimers Dement..

[4] Knopman D.S., Amieva H., Petersen R.C., Chételat G., Holtzman D.M., Hyman B.T., Nixon R.A., Jones D.T. (2021). Alzheimer disease. Nat. Rev. Dis. Prim..

[5] Ballard C., Aarsland D., Cummings J., O’Brien J., Mills R., Molinuevo J.L., Fladby T., Williams G., Doherty P., Corbett A. (2020). Drug repositioning and repurposing for Alzheimer disease. Nat. Rev. Neurol..

[6] Avila J., Perry G.A. (2021). Multilevel View of the Development of Alzheimer’s Disease. Neuroscience.

[7] McMurtray A., Clark D.G., Christine D., Mendez M.F. (2006). Early-onset dementia: Frequency and causes compared to late-onset dementia. Dement. Geriatr. Cogn. Disord..

[8] Irie F., Fitzpatrick A.L., Lopez O.L., Kuller L.H., Peila R., Newman A.B., Launer L.J. (2008). Enhanced risk for Alzheimer disease in persons with type 2 diabetes and APOE epsilon4: The Cardiovascular Health Study Cognition Study. Arch. Neurol..

[9] Chen Y., Hong T., Chen F., Sun Y., Wang Y., Cui L. (2021). Interplay between Microglia and Alzheimer’s Disease-Focus on the Most Relevant Risks: APOE Genotype, Sex and Age. Front. Aging Neurosci..

[10] Serrano-Pozo A., Das S., Hyman B.T. (2021). APOE and Alzheimer’s disease: Advances in genetics, pathophysiology, and therapeutic approaches. Lancet Neurol..

[11] Emrani S., Arain H.A., De Marshall C., Nuriel T. (2020). APOE4 is associated with cognitive and pathological heterogeneity in patients with Alzheimer’s disease: A systematic review. Alzheimers Res. Ther..

[12] Yamazaki Y., Zhao N., Caulfield T.R., Liu C.C., Bu G. (2019). Apolipoprotein E and Alzheimer disease: Pathobiology and targeting strategies. Nat. Rev. Neurol..

[13] Qin Q., Teng Z., Liu C., Li Q., Yin Y., Tang Y. (2021). TREM2, microglia, and Alzheimer’s disease. Mech. Ageing Dev..

[14] Xue F., Du H. (2021). TREM2 Mediates Microglial Anti-Inflammatory Activations in Alzheimer’s Disease: Lessons Learned from Transcriptomics. Cells.

[15] Li L., Cavuoto M., Biddiscombe K., Pike K.E. (2020). Diabetes Mellitus Increases Risk of Incident Dementia in APOEɛ4 Carriers: A Meta-Analysis. J. Alzheimers Dis..

[16] Rojas-Gutierrez E., Muñoz-Arenas G., Treviño S., Espinosa B., Chavez R., Rojas K., Flores G., Díaz A., Guevara J. (2017). Alzheimer’s disease and metabolic syndrome: A link from oxidative stress and inflammation to neurodegeneration. Synapse.

[17] De la Monte S.M., Tong M., Lester-Coll N., Plater M., Wands J.R. (2006). Therapeutic rescue of neurodegeneration in experimental type 3 diabetes: Relevance to Alzheimer’s disease. J. Alzheimers Dis..

[18] de la Monte S.M. (2019). The Full Spectrum of Alzheimer’s Disease Is Rooted in Metabolic Derangements That Drive Type 3 Diabetes. Adv. Exp. Med. Biol..

[19] de la Monte S.M., Tong M., Wands J.R. (2018). The 20-Year Voyage Aboard the Journal of Alzheimer’s Disease: Docking at ‘Type 3 Diabetes’, Environmental/Exposure Factors, Pathogenic Mechanisms, and Potential Treatments. J. Alzheimers Dis..

[20] de la Monte S.M. (2014). Type 3 diabetes is sporadic Alzheimer’s disease: Mini-review. Eur. Neuropsychopharmacol..

[21] Gudala K., Bansal D., Schifano F., Bhansali A. (2013). Diabetes mellitus and risk of dementia: A meta-analysis of prospective observational studies. J. Diabetes Investig..

[22] Burillo J., Marqués P., Jiménez B., González-Blanco C., Benito M., Guillén C. (2021). Insulin Resistance and Diabetes Mellitus in Alzheimer’s Disease. Cells.

[23] Carranza-Naval M.J., Vargas-Soria M., Hierro-Bujalance C., Baena-Nieto G., Garcia-Alloza M., Infante-Garcia C., Del Marco A. (2021). Alzheimer’s Disease and Diabetes: Role of Diet, Microbiota and Inflammation in Preclinical Models. Biomolecules.

[24] Rosenfeld J.E. (2014). Metformin and Alzheimer’s disease risk. Am. J. Psychiatry.

[25] Vinuesa A., Pomilio C., Gregosa A., Bentivegna M., Presa J., Bellotto M., Saravia F., Beauquis J. (2021). Inflammation and Insulin Resistance as Risk Factors and Potential Therapeutic Targets for Alzheimer’s Disease. Front. Neurosci..

[26] Sun Y., Ma C., Sun H., Wang H., Peng W., Zhou Z., Wang H., Pi C., Shi Y., He X. (2020). Metabolism: A Novel Shared Link between Diabetes Mellitus and Alzheimer’s Disease. J. Diabetes Res..

[27] Ott A., Stolk R.P., Hofman A., van Harskamp F., Grobbee D.E., Breteler M.M. (1996). Association of diabetes mellitus and dementia: The Rotterdam Study. Diabetologia.

[28] Ott A., Stolk R.P., van Harskamp F., Pols H.A., Hofman A., Breteler M.M. (1999). Diabetes mellitus and the risk of dementia: The Rotterdam Study. Neurology.

[29] Salameh T.S., Rhea E.M., Banks W.A., Hanson A.J. (2016). Insulin resistance, dyslipidemia, and apolipoprotein E interactions as mechanisms in cognitive impairment and Alzheimer’s disease. Exp. Biol. Med..

[30] Matsuzaki T., Sasaki K., Tanizaki Y., Hata J., Fujimi K., Matsui Y., Sekita A., Suzuki S.O., Kanba S., Kiyohara Y. (2010). Insulin resistance is associated with the pathology of Alzheimer disease: The Hisayama study. Neurology.

[31] Willette A.A., Bendlin B.B., Starks E.J., Birdsill A.C., Johnson S.C., Christian B.T., Okonkwo O.C., La Rue A., Hermann B.P., Koscik R.L. (2015). Association of insulin resistance with cerebral glucose uptake in late middle-aged adults at risk for Alzheimer disease. JAMA Neurol..

[32] Willette A.A., Xu G., Johnson S.C., Birdsill A.C., Jonaitis E.M., Sager M.A., Hermann B.P., La Rue A., Asthana S., Bendlin B.B. (2013). Insulin resistance, brain atrophy, and cognitive performance in late middle-aged adults. Diabetes Care.

[33] Rebelos E., Rinne J.O., Nuutila P., Ekblad L.L. (2021). Brain Glucose Metabolism in Health, Obesity, and Cognitive Decline-Does Insulin Have Anything to Do with It? A Narrative Review. J. Clin. Med..

[34] Berlanga-Acosta J., Guillén-Nieto G., Rodríguez-Rodríguez N., Bringas-Vega M.L., García-Del-Barco-Herrera D., Berlanga-Saez J.O., García-Ojalvo A., Valdés-Sosa M.J., Valdés-Sosa P.A. (2020). Insulin Resistance at the Crossroad of Alzheimer Disease Pathology: A Review. Front. Endocrinol..

[35] Biessels G.J., Staekenborg S., Brunner E., Brayne C., Scheltens P. (2006). Risk of dementia in diabetes mellitus: A systematic review. Lancet Neurol..

[36] Talbot K., Wang H.Y., Kazi H., Han L.Y., Bakshi K.P., Stucky A., Fuino R.L., Kawaguchi K.R., Samoyedny A.J., Wilson R.S. (2012). Demonstrated brain insulin resistance in Alzheimer’s disease patients is associated with IGF-1 resistance, IRS-1 dysregulation, and cognitive decline. J. Clin. Investig..

[37] Arvanitakis Z., Wang H.Y., Capuano A.W., Khan A., Taïb B., Anokye-Danso F., Schneider J.A., Bennett D.A., Ahima R.S., Arnold S.E. (2020). Brain Insulin Signaling, Alzheimer Disease Pathology, and Cognitive Function. Ann. Neurol..

[38] Barber T.M., Kyrou I., Randeva H.S., Weickert M.O. (2021). Mechanisms of Insulin Resistance at the Crossroad of Obesity with Associated Metabolic Abnormalities and Cognitive Dysfunction. Int. J. Mol. Sci..

[39] Barnes D.E., Yaffe K. (2011). The projected effect of risk factor reduction on Alzheimer’s disease prevalence. Lancet Neurol..

[40] Carvalho C., Cardoso S.M., Correia S.C., Moreira P.I. (2019). Tortuous Paths of Insulin Signaling and Mitochondria in Alzheimer’s Disease. Adv. Exp. Med. Biol..

[41] De Felice F.G. (2013). Connecting type 2 diabetes to Alzheimer’s disease. Expert Rev. Neurother..

[42] De Felice F.G., Benedict C. (2015). A Key Role of Insulin Receptors in Memory. Diabetes.

[43] Kulstad J.J., Green P.S., Cook D.G., Watson G.S., Reger M.A., Baker L.D., Plymate S.R., Asthana S., Rhoads K., Mehta P.D. (2006). Craft Differential modulation of plasma beta-amyloid by insulin in patients with Alzheimer disease. Neurology.

[44] Lacor P.N. (2007). Advances on the understanding of the origins of synaptic pathology in AD. Curr. Genom..

[45] Lacor P.N., Buniel M.C., Furlow P.W., Clemente A.S., Velasco P.T., Wood M., Viola K.L., Klein W.L. (2007). Abeta oligomer-induced aberrations in synapse composition, shape, and density provide a molecular basis for loss of connectivity in Alzheimer’s disease. J. Neurosci..

[46] Viola K.L., Klein W.L. (2015). Amyloid β oligomers in Alzheimer’s disease pathogenesis, treatment, and diagnosis. Acta Neuropathol..

[47] Cline E.N., Bicca M.A., Viola K.L., Klein W.L. (2018). The amyloid- oligomer hypothesis: Beginning of the third decade. J. Alzheimers Dis..

[48] Li S., Selkoe D.J. (2020). A mechanistic hypothesis for the impairment of synaptic plasticity by soluble Abeta oligomers from Alzheimer’s brain. J. Neurochem..

[49] Ferreira S.T., Lourenco M.V., Oliveira M.M., De Felice F.G. (2015). Soluble amyloid-β oligomers as synaptotoxins leading to cognitive impairment in Alzheimer’s disease. Front. Cell Neurosci..

[50] Fishel M.A., Watson G.S., Montine T.J., Wang Q., Green P.S., Kulstad J.J., Cook D.G., Peskind E.R., Baker L.D., Goldgaber D. (2005). Hyperinsulinemia provokes synchronous increases in central inflammation and beta-amyloid in normal adults. Arch. Neurol..

[51] Takeda S., Sato N., Uchio-Yamada K., Sawada K., Kunieda T., Takeuchi D., Kurinami H., Shinohara M., Rakugi H., Morishita R. (2009). Elevation of plasma beta-amyloid level by glucose loading in Alzheimer mouse models. Biochem. Biophys. Res. Commun..

[52] Xie L., Helmerhorst E., Taddei K., Plewright B., Van Bronswijk W., Martins R. (2002). Alzheimer’s beta-amyloid peptides compete for insulin binding to the insulin receptor. J. Neurosci..

[53] Zhang Y., Zhou B., Zhang F., Wu J., Hu Y., Liu Y., Zhai Q. (2012). Amyloid-β induces hepatic insulin resistance by activating JAK2/STAT3/SOCS-1 signaling pathway. Diabetes.

[54] Zhang Y., Zhou B., Deng B., Zhang F., Wu J., Wang Y., Le Y., Zhai Q. (2013). Amyloid-β induces hepatic insulin resistance in vivo via JAK2. Diabetes.

[55] de la Monte S.M., Tong M., Daiello L.A., Ott B.R. (2019). Early-Stage Alzheimer’s Disease Is Associated with Simultaneous Systemic and Central Nervous System Dysregulation of Insulin-Linked Metabolic Pathways. J. Alzheimers Dis..

[56] El Massry M., Alaeddine L.M., Ali L., Saad C., Eid A.A. (2021). Metformin: A Growing Journey from Glycemic Control to the Treatment of Alzheimer’s Disease and Depression. Curr. Med. Chem..

[57] Plucińska K., Dekeryte R., Koss D., Shearer K., Mody N., Whitfield P.D., Doherty M.K., Mingarelli M., Welch A., Riedel G. (2016). Neuronal human BACE1 knockin induces systemic diabetes in mice. Diabetologia.

[58] Gratuze M., Joly-Amado A., Vieau D., Buée L., Blum D. (2018). Mutual Relationship between Tau and Central Insulin Signalling: Consequences for AD and Tauopathies?. Neuroendocrinology.

[59] Marciniak E., Leboucher A., Caron E., Ahmed T., Tailleux A., Dumont J., Issad T., Gerhardt E., Pagesy P., Vileno M. (2017). Tau deletion promotes brain insulin resistance. J. Exp. Med..

[60] Rodriguez-Rodriguez P., Sandebring-Matton A., Merino-Serrais P., Parrado-Fernandez C., Rabano A., Winblad B., Ávila J., Ferrer I., Cedazo-Minguez A. (2017). Tau hyperphosphorylation induces oligomeric insulin accumulation and insulin resistance in neurons. Brain.

[61] Yarchoan M., Toledo J.B., Lee E.B., Arvanitakis Z., Kazi H., Han L.Y., Louneva N., Lee V.M., Kim S.F., Trojanowski J.Q. (2014). Abnormal serine phosphorylation of insulin receptor substrate 1 is associated with tau pathology in Alzheimer’s disease and tauopathies. Acta Neuropathol..

[62] Gonçalves R.A., Wijesekara N., De Felice F.G. (2019). The Link between Tau and Insulin Signaling: Implications for Alzheimer’s Disease and Other Tauopathies. Front. Cell Neurosci..

[63] Wang Y., Mandelkow E. (2016). Tau in physiology and pathology. Nat. Rev. Neurosci..

[64] Rena G., Hardie D.G., Pearson E.R. (2017). The mechanisms of action of metformin. Diabetologia.

[65] Markowicz-Piasecka M., Sikora J., Szydłowska A., Skupień A., Mikiciuk-Olasik E., Huttunen K.M. (2017). Metformin–A future therapy for neurodegenerative diseases. Pharm. Res..

[66] Chaudhari K., Reynolds C.D., Yang S.H. (2020). Metformin and cognition from the perspectives of sex, age, and disease. Geroscience.

[67] Bendlin B.B. (2019). Antidiabetic therapies and Alzheimer disease. Dialogues Clin. Neurosci..

[68] Lv W.S., Wen J.P., Li L., Sun R.X., Wang J., Xian Y.X., Cao C.X., Wang Y.L., Gao Y.Y. (2012). The effect of metformin on food intake and its potential role in hypothalamic regulation in obese diabetic rats. Brain Res..

[69] Syal C., Kosaraju J., Hamilton L., Aumont A., Chu A., Sarma S.N., Thomas J., Seegobin M., Dilworth F.J., He L. (2020). Dysregulated expression of monoacylglycerol lipase is a marker for anti-diabetic drug metformin-targeted therapy to correct impaired neurogenesis and spatial memory in Alzheimer’s disease. Theranostics.

[70] Chen M., Huang N., Liu J., Huang J., Shi J., Jin F. (2021). AMPK: A bridge between diabetes mellitus and Alzheimer’s disease. Behav. Brain Res..

[71] Mostafa D.K., Ismail C.A., Ghareeb D.A. (2016). Differential metformin dose-dependent effects on cognition in rats: Role of Akt. Psychopharmacology.

[72] Kodali M., Attaluri S., Madhu L.N., Shuai B., Upadhya R., Gonzalez J.J., Rao X., Shetty A.K. (2021). Metformin treatment in late middle age improves cognitive function with alleviation of microglial activation and enhancement of autophagy in the hippocampus. Aging Cell.

[73] Laurijssens B., Aujard F., Rahman A. (2013). Animal models of Alzheimer’s disease and drug development. Drug. Discov. Today Technol..

[74] Nakai T., Yamada K., Mizoguchi H. (2021). Alzheimer’s Disease Animal Models: Elucidation of Biomarkers and Therapeutic Approaches for Cognitive Impairment. Int. J. Mol. Sci..

[75] Correia S., Carvalho C., Santos M.S., Proença T., Nunes E., Duarte A.I., Monteiro P., Seiça R., Oliveira C.R., Moreira P.I. (2008). Metformin protects the brain against the oxidative imbalance promoted by type 2 diabetes. Med. Chem..

[76] Chen Y., Zhao S., Fan Z., Li Z., Zhu Y., Shen T., Li K., Yan Y., Tian J., Liu Z. (2021). Metformin attenuates plaque-associated tau pathology and reduces amyloid-β burden in APP/PS1 mice. Alzheimer Res. Ther..

[77] Trujillo-Estrada L., Sanchez-Mejias E., Sanchez-Varo R., Garcia-Leon J.A., Nuñez-Diaz C., Davila J.C., Vitorica J., LaFerla F.M., Moreno-Gonzalez I., Gutierrez A. (2021). Animal and Cellular Models of Alzheimer’s Disease: Progress, Promise, and Future Approaches. Neuroscientist.

[78] Bomfim T.R., Forny-Germano L., Sathler L.B., Brito-Moreira J., Houzel J.C., Decker H., Silverman M.A., Kazi H., Melo H.M., McClean P.L. (2012). An anti-diabetes agent protects the mouse brain from defective insulin signaling caused by Alzheimer’s disease- associated Aβ oligomers. J. Clin. Investig..

[79] Obafemi T.O., Olasehinde O.R., Olaoye O.A., Jaiyesimi K.F., Adewumi F.D., Adewale O.B., Afolabi B.A. (2020). Metformin/Donepezil combination modulates brain antioxidant status and hippocampal endoplasmic reticulum stress in type 2 diabetic rats. J. Diabetes Metab. Disord..

[80] Grieb P. (2016). Intracerebroventricular Streptozotocin Injections as a Model of Alzheimer’s Disease: In Search of a Relevant Mechanism. Mol. Neurobiol..

[81] Esmaeili M.H., Esmaeili M. (2016). Metformin improves learning and memory in streptozotocin-induced rat model of sporadic Alzheimer’s disease. Indian J. Fund. App. Life Sci..

[82] Ou Z., Kong X., Sun X., He X., Zhang L., Gong Z., Huang J., Xu B., Long D., Li J. (2018). Metformin treatment prevents amyloid plaque deposition and memory impairment in APP/PS1 mice. Brain Behav. Immun..

[83] Oliveira W.H., Nunes A.K., França M.E., Santos L.A., Lós D.B., Rocha S.W., Barbosa K.P., Rodrigues G.B., Peixoto C.A. (2016). Effects of metformin on inflammation and short-term memory in streptozotocin-induced diabetic mice. Brain Res..

[84] Pilipenko V., Narbute K., Pupure J., Langrate I.K., Muceniece R., Kluša V. (2020). Neuroprotective potential of antihyperglycemic drug metformin in streptozotocin-induced rat model of sporadic Alzheimer’s disease. Eur. J. Pharmacol..

[85] Salkovic-Petrisic M., Knezovic A., Hoyer S., Riederer P. (2013). What have we learned from the streptozotocin-induced animal model of sporadic Alzheimer’s disease, about the therapeutic strategies in Alzheimer’s research. J. Neural Transm..

[86] Plaschke K., Kopitz J., Siegelin M., Schliebs R., Salkovic-Petrisic M., Riederer P., Hoyer S. (2010). Insulin-resistant brain state after intracerebroventricular streptozotocin injection exacerbates Alzheimer-like changes in Tg2576 AbetaPP-overexpressing mice. J. Alzheimers Dis..

[87] Grünblatt E., Salkovic-Petrisic M., Osmanovic J., Riederer P., Hoyer S.J. (2007). Brain insulin system dysfunction in streptozotocin intracerebroventricularly treated rats generates hyperphosphorylated tau protein. J. Neurochem..

[88] Salkovic-Petrisic M., Hoyer S.J. (2007). Central insulin resistance as a trigger for sporadic Alzheimer-like pathology: An experimental approach. Neuropsychiatr. Disord. Integr. Approach.

[89] Saffari P.M., Alijanpour S., Takzaree N., Sahebgharani M., Etemad-Moghadam S., Noorbakhsh F., Partoazar A. (2020). Metformin loaded phosphatidylserine nanoliposomes improve memory deficit and reduce neuroinflammation in streptozotocin-induced Alzheimer’s disease model. Life Sci..

[90] Ditacchio K.A., Heinemann S.F., Dziewczapolski G. (2015). Metformin treatment alters memory function in a mouse model of Alzheimer’s disease. J. Alzheimers Dis..

[91] Farr S.A., Roesler E., Niehoff M.L., Roby D.A., McKee A., Morley J.E. (2019). Metformin improves learning and memory in the SAMP8 mouse model of Alzheimer’s disease. J. Alzheimers Dis..

[92] Bao J., Mahaman Y.A.R., Liu R., Wang J.Z., Zhang Z., Zhang B., Wang X. (2017). Sex Differences in the Cognitive and Hippocampal Effects of Streptozotocin in an Animal Model of Sporadic AD. Front. Aging Neurosci..

[93] Lu X.Y., Huang S., Chen Q.B., Zhang D., Li W., Ao R., Leung F.C., Zhang Z., Huang J., Tang Y. (2020). Metformin Ameliorates Abeta Pathology by Insulin-Degrading Enzyme in a Transgenic Mouse Model of Alzheimer’s disease. Oxid. Med. Cell Longev..

[94] Katila N., Bhurtel S., Park P.H., Hong J.T., Choi D.Y. (2020). Activation of AMPK/aPKCζ/CREB pathway by metformin is associated with upregulation of GDNF and dopamine. Biochem. Pharmacol..

[95] Aksoz E., Gocmez S.S., Sahin T.D., Aksit D., Aksit H., Utkan T. (2019). The protective effect of metformin in scopolamine-induced learning and memory impairment in rats. Pharmacol. Rep..

[96] Asadbegi M., Yaghmaei P., Salehi I., Ebrahim-Habibi A., Komaki A. (2016). Neuroprotective effects of metformin against Aβ-mediated inhibition of long-term potentiation in rats fed a high-fat diet. Brain Res. Bull..

[97] Chin-Hsiao T. (2019). Metformin and the Risk of Dementia in Type 2 Diabetes Patients. Aging Dis..

[98] Shi Q., Liu S., Fonseca V.A., Thethi T.K., Shi L. (2019). Effect of metformin on neurodegenerative disease among elderly adult US veterans with type 2 diabetes mellitus. BMJ Open.

[99] Luchsinger J.A., Perez T., Chang H., Mehta P., Steffener J., Pradabhan G., Ichise M., Manly J., Devanand D.P., Bagiella E. (2016). Metformin in amnestic mild cognitive impairment: Results of a pilot randomized placebo controlled clinical trial. J. Alzheimers Dis..

[100] Luchsinger J.A., Ma Y., Christophi C.A., Florez H., Golden S.H., Hazuda H., Crandall J., Venditti E., Watson K., Jeffries S. (2017). Diabetes Prevention Program Research Group. Metformin, Lifestyle Intervention, and Cognition in the Diabetes Prevention Program Outcomes Study. Diabetes Care.

[101] Koenig A.M., Mechanic-Hamilton D., Xie S.X., Combs M.F., Cappola A.R., Xie L., Detre J.A., Wolk D.A., Arnold S.E. (2017). Effects of the insulin sensitizer metformin in Alzheimer disease: Pilot data from a randomized placebo-controlled crossover study. Alzheimer Dis. Assoc. Disord..

[102] Madhu L.N., Kodali M., Shetty A.K. (2022). Promise of metformin for preventing age-related cognitive dysfunction. Neural Regen Res..

[103] Scherrer J.F., Morley J.E., Salas J., Floyd J.S., Farr S.A., Dublin S. (2019). Association between Metformin Initiation and Incident Dementia among African American and White Veterans Health Administration Patients. Ann. Fam. Med..

[104] Sluggett J.K., Koponen M., Bell J.S., Taipale H., Tanskanen A., Tiihonen J., Uusitupa M., Tolppanen A.M., Hartikainen S. (2020). Metformin and Risk of Alzheimer’s Disease among Community-Dwelling people with Diabetes: A National Case-Control Study. J. Clin. Endocrinol. Metab..

[105] Koo B.K., Kim L.K., Lee J.Y., Moon M.K. (2019). Taking metformin and cognitive function change in older patients with diabetes. Geriatr. Gerontol. Int..

[106] Makkar S., Crawford J.D., Kochan N.A., Wen W., Draper B., Trollor J.N., Brodaty H., Sachdev P.S. (2020). Metformin Use Is Associated with Slowed Cognitive Decline and Reduced Incident Dementia in Older Adults with Type 2 Diabetes: The Sydney Memory and Ageing Study. Diabetes Care.

[107] Campbell J.M., Stephenson M.D., de Courten B., Chapman I., Bellman S.M., Aromataris E. (2018). Metformin Use Associated with Reduced Risk of Dementia in Patients with Diabetes: A Systematic Review and Meta-Analysis. J. Alzheimers Dis..

[108] Ramamurthy S., Ronnett G. (2012). AMP-activated protein kinase (AMPK) and energy-sensing in the brain. Exp. Neurobiol..

[109] Domise M., Vingtdeux V. (2016). AMPK in Neurodegenerative Diseases. Exp. Suppl..

[110] Seixas da Silva G.S., Melo H.M., Lourenco M.V., Lyra E.S.N.M., de Carvalho M.B., Alves-Leon S.V., de Souza J.M., Klein W.L., da-Silva W.S., Ferreira S.T. (2017). Amyloid-β oligomers transiently inhibit AMP-activated kinase and cause metabolic defects in hippocampal neurons. J. Biol. Chem..

[111] Culmsee C., Monnig J., Kemp B.E., Mattson M.P. (2001). AMP-activated kinase is highly expressed in neurons in the developing rat brain and promotes neuronal survival following glucose deprivation. J. Mol. Neurosci..

[112] Gupta A., Bisht B., Dey C.S. (2011). Peripheral insulin-sensitizer drug metformin ameliorates neuronal insulin resistance and Alzheimer’s-like changes. Neuropharmacology.

[113] Wilson C.M., Magnaudeix A., Yardin C., Terro F. (2014). Autophagy dysfunction and its link to Alzheimer’s disease and type II diabetes mellitus. CNS Neurol. Disord. Drug Targets.

[114] Cantó C., Gerhart-Hines Z., Feige J.N., Lagouge M., Noriega L., Milne J.C., Elliott P.J., Puigserver P., Auwerx J.C. (2009). AMPK regulates energy expenditure by modulating NAD+ metabolism and SIRT1 activity. Nature.

[115] Cantó C., Auwerx J. (2010). AMP-activated protein kinase and its downstream transcriptional pathways. Cell Mol. Life Sci..

[116] Wang L., Li N., Shi F.X., Xu W.Q., Cao Y., Lei Y., Wang J.Z., Tian Q., Zhou X.W. (2020). Upregulation of AMPK Ameliorates Alzheimer’s Disease-Like Tau Pathology and Memory Impairment. Mol. Neurobiol..

[117] Vingtdeux V., Davies P., Dickson D.W., Marambaud P. (2011). AMPK is abnormally activated in tangle- and pre-tangle-bearing neurons in Alzheimer’s disease and other tauopathies. Acta Neuropathol..

[118] Cunnane S.C., Trushina E., Morland C., Prigione A., Casadesus G., Andrews Z.B., Beal M.F., Bergersen L.H., Brinton R.D., de la Monte S. (2020). Brain energy rescue: An emerging therapeutic concept for neurodegenerative disorders of ageing. Nat. Rev. Drug Discov..

[119] Kickstein E., Krauss S., Thornhill P., Rutschow D., Zeller R., Sharkey J., Williamson R., Fuchs M., Köhler A., Glossmann H. (2010). Biguanide metformin acts on tau phosphorylation via mTOR/protein phosphatase 2A (PP2A) signaling. Proc. Natl. Acad. Sci. USA.

[120] Domise M., Didier S., Marinangeli C., Zhao H., Chandakkar P., Buée L., Viollet B., Davies P., Marambaud P., Vingtdeux V. (2016). AMP-activated protein kinase modulates tau phosphorylation and tau pathology in vivo. Sci. Rep..

[121] Ma X., Xiao W., Li H., Pang P., Xue F., Wan L., Pei L., Yan H. (2021). Metformin restores hippocampal neurogenesis and learning and memory via regulating gut microbiota in the obese mouse model. Brain Behav. Immun..

[122] Peng Y., Gao P., Shi L., Chen L., Liu J., Long J. (2020). Central and peripheral metabolic defects contribute to the pathogenesis of alzheimer’s disease: Targeting mitochondria for diagnosis and prevention. Antioxid. Redox Signal..

[123] Swerdlow R.H. (2018). Mitochondra and mitochondrial cascades in alzheimer’s disease. J. Alzheimers Dis..

[124] Chung M.-M., Chen Y.L., Pei D., Cheng Y.C., Sun B., Nicol C.J., Yen C.H., Chen H.M., Liang Y.J., Chiang M.C. (2015). The neuroprotective role of metformin in advanced glycation end product treated human neural stem cells is AMPK-dependent. Biochim. Biophys. Acta.

[125] Gouveia A., Hsu K., Niibori Y., Seegobin M., Cancino G.I., He L., Wondisford F.E., Bennett S., Lagace D., Frankland P.W. (2016). The aPKC-CBP Pathway Regulates Adult Hippocampal Neurogenesis in an Age-Dependent Manner. Stem. Cell Rep..

[126] Tanokashira D., Kurata E., Fukuokaya W., Kawabe K., Kashiwada M., Takeuchi H., Nakazato M., Taguchi A. (2018). Metformin treatment ameliorates diabetes-associated decline in hippocampal neurogenesis and memory via phosphorylation of insulin receptor substrate 1. FEBS Open Biol..

[127] Rajangam J., Bhatt S., Krishnan N., Sammeta M., Joshna L. (2020). Influence of Metformin on learning and memory in experimental Amnesia model in Mice. Ann. Alzheimers Dement. Care.

[128] Boccardi V., Murasecco I., Mecocci P. (2019). Diabetes drugs in the fight against Alzheimer’s disease. Ageing Res. Rev..

[129] Muñoz-Jiménez M., Zaarkti A., García-Arnés J.A., García-Casares N. (2020). Antidiabetic Drugs in Alzheimer’s disease and Mild Cognitive Impairment: A Systematic Review. Dement. Geriatr. Cogn. Disord..

[130] Zhu X., Shen J., Feng S., Huang C., Liu Z., Sun Y.E., Liu H. (2020). Metformin improves cognition of aged mice by promoting cerebral angiogenesis and neurogenesis. Aging.

[131] Chen W.B., Chen J., Liu Z.Y., Luo B., Zhou T., Fei E.K. (2020). Metformin Enhances Excitatory Synaptic Transmission onto Hippocampal CA1 Pyramidal Neurons. Brain Sci..

[132] Grillo C.A., Piroli G.G., Lawrence R.C., Wrighten S.A., Green A.J., Wilson S.P., Sakai R.R., Kelly S.J., Wilson M.A., Mott D.D. (2015). Hippocampal Insulin Resistance Impairs Spatial Learning and Synaptic Plasticity. Diabetes.

[133] Li J., Deng J., Sheng W., Zuo Z. (2012). Metformin attenuates Alzheimer’s disease-like neuropathology in obese, leptin-resistant mice. Pharm. Biochem. Behav..

[134] Boros B.D., Greathouse K.M., Gentry E.G., Curtis K.A., Birchall E.L., Gearing M., Herskowitz J.H. (2017). Dendritic spines provide cognitive resilience against Alzheimer’s disease. Ann. Neurol..

[135] Boros B.D., Greathouse K.M., Gearing M., Herskowitz J.H. (2019). Dendritic spine remodeling accompanies Alzheimer’s disease pathology and genetic susceptibility in cognitively normal aging. Neurobiol. Aging.

[136] Walker C.K., Herskowitz J.H. (2020). Dendritic spines: Mediators of cognitive resilience in aging and Alzheimer’s disease. Neuroscientist.

[137] Morrison J.H., Baxter M.G. (2014). Synaptic health. JAMA Psychiatry.

[138] Dumitriu D., Hao J., Hara Y., Kaufmann J., Janssen W.G., Lou W., Rapp P.R., Morrison J.H. (2010). Selective changes in thin spine density and morphology in monkey prefrontal cortex correlate with aging-related cognitive impairment. J. Neurosci..

[139] Pereira A.C., Lambert H.K., Grossman Y.S., Dumitriu D., Waldman R., Jannetty S.K., Calakos K., Janssen W.G., McEwen B.S., Morrison J.H. (2014). Glutamatergic regulation prevents hippocampal-dependent age-related cognitive decline through dendritic spine clustering. Proc. Natl. Acad. Sci. USA.

[140] Scheff S.W., Price D., Schmitt F.A., Mufson E.J. (2006). Hippocampal synaptic loss in early Alzheimer’s disease and mild cognitive impairment. Neurobiol. Aging.

[141] Scheff S.W., Neltner J.H., Nelson P.T. (2014). Is synaptic loss a unique hallmark of Alzheimer’s disease?. Biochem. Pharmacol..

[142] Cardoso S., Moreira P.I. (2020). Antidiabetic drugs for Alzheimer’s and Parkinson’s diseases: Repurposing insulin, metformin, and thiazolidinediones. Int. Rev. Neurobiol..

[143] Soo S.K., Rudich P.D., Traa A., Harris-Gauthier N., Shields H.J., Van Raamsdonk J.M. (2020). Compounds that extend longevity are protective in neurodegenerative diseases and provide a novel treatment strategy for these devastating disorders. Mech. Ageing Dev..

[144] Wang Y., Zhao J., Guo F.L., Gao X., Xie X., Liu S., Yang X., Yang X., Zhang L., Ye Y. (2020). Metformin Ameliorates Synaptic Defects in a Mouse Model of AD by Inhibiting Cdk5 Activity. Front. Cell Neurosci..

[145] Liu W., Zhou Y., Liang R., Zhang Y. (2019). Inhibition of cyclin-dependent kinase 5 activity alleviates diabetes-related cognitive deficits. FASEB J..

[146] Cai H.B., Fan Z.Z., Tian T., Li Z.C., Zhao C.C., Guo W.T., Ge Z.M. (2020). Diabetes-induced H3K9 Hyperacetylation Promotes Development of Alzheimer’s Disease through CDK5. J. Alzheimers Dis..

[147] Ha J.S., Yeom Y.S., Jang J.H., Kim Y.H., Im J.I., Kim I.S., Yang S.J. (2019). Anti-inflammatory Effects of Metformin on Neuro-inflammation and NLRP3 Inflammasome Activation in BV-2 Microglial Cells. Biomed. Sci. Lett..

[148] Liu Y., Tang G., Zhang Z., Wang Y., Yang G.Y. (2014). Metformin promotes focal angiogenesis and neurogenesis in mice following middle cerebral artery occlusion. Neurosci. Lett..

[149] Hettich M.M., Matthes F., Ryan D.P., Griesche N., Schröder S., Dorn S., Krauβ S., Ehninger D. (2014). The anti-diabetic drug metformin reduces BACE1 protein level by interfering with the MID1 complex. PLoS ONE.

[150] Hasanpour Dehkordi A., Abbaszadeh A., Mir S., Hasanvand A. (2019). Metformin and its anti-inflammatory and anti-oxidative effects; new concepts. J. Renal Inj. Prev..

[151] Miziak B., Błaszczyk B., Czuczwar S.J. (2021). Some Candidate Drugs for Pharmacotherapy of Alzheimer’s Disease. Pharmaceuticals.

[152] Wareski P., Vaarmann A., Choubey V., Safiulina D., Liiv J., Kuum M., Kaasik A. (2009). PGC-1{alpha} and PGC-1{beta} regulate mitochondrial density in neurons. Biol. Chem..

[153] Fatt M., Hsu K., He L., Wondisford F., Miller F.D., Kaplan D.R., Wang J. (2015). Metformin Acts on Two Different Molecular Pathways to Enhance Adult Neural Precursor Proliferation/Self-Renewal and Differentiation. Stem. Cell Rep..

[154] Dziedzic A., Saluk-Bijak J., Miller E., Bijak M. (2020). Metformin as a Potential Agent in the Treatment of Multiple Sclerosis. Int. J. Mol. Sci..

[155] Esmaeilnejad S., Semnanian S., Javan M. (2021). Metformin Protects Myelin from Degeneration in A Mouse Model of Iysophosphatidylcholine-Induced Demyelination in The Optic Chiasm. Cell J..

[156] Corcoran C., Jacobs T.F. (2021). Metformin. StatPearls [Internet]. Treasure Island (FL).

